# Amino Acids in Nine Ligand-Prefer Ramachandran Regions

**DOI:** 10.1155/2015/757495

**Published:** 2015-09-29

**Authors:** Chen Cao, Lincong Wang, Xiaoyang Chen, Shuxue Zou, Guishen Wang, Shutan Xu

**Affiliations:** ^1^College of Computer Science and Technology, Jilin University, Changchun, Jilin, China; ^2^Key Laboratory of Symbol Computation and Knowledge Engineering of the Ministry of Education, Jilin University, Changchun, Jilin, China

## Abstract

Several secondary structures, such as *π*-helix and left-handed helix, have been frequently identified at protein ligand-binding sites. A secondary structure is considered to be constrained to a specific region of dihedral angles. However, a comprehensive analysis of the correlation between main chain dihedral angles and ligand-binding sites has not been performed. We undertook an extensive analysis of the relationship between dihedral angles in proteins and their distance to ligand-binding sites, frequency of occurrence, molecular potential energy, amino acid composition, van der Waals contacts, and hydrogen bonds with ligands. The results showed that the values of dihedral angles have a strong preference for ligand-binding sites at certain regions in the Ramachandran plot. We discovered that amino acids preceding the ligand-prefer *ϕ*/*ψ* box residues are exposed more to solvents, whereas amino acids following ligand-prefer *ϕ*/*ψ* box residues form more hydrogen bonds and van der Waals contacts with ligands. Our method exhibited a similar performance compared with the program Ligsite-csc for both ligand-bound structures and ligand-free structures when just one ligand-binding site was predicted. These results should be useful for the prediction of protein ligand-binding sites and for analysing the relationship between structure and function.

## 1. Introduction

The two main chain dihedral torsion angles that describe the rotations of the polypeptide backbone around the bonds between N-C*α* (*ϕ*) and C*α*-C (*ψ*) were identified by Ramakrishnan and Ramachandran [1]. These two torsion angles provide flexibility for the polypeptide backbone to adopt a fixed fold because the third possible torsion angle between C-N (*Ω*) is almost flat and fixed at 180°. The application of these two torsion angles, which describe the protein backbone conformation approach, has been widespread. The Ramachandran plot, which has remained unchanged for fifty years, provides a simple view of the distribution of the two torsion angles in protein structures [2]. The two dihedral torsion angles have also been applied in fields such as secondary structure assignment and protein structure refinement [3–5].

Secondary structure refers to highly regular local sub-substructures in proteins, of which *α*-helix and *β*-sheet are the two main types. In 1951, Pauling et al. first defined these secondary structures using the hydrogen pattern between the main chain backbone amino (NH) and carbonyl (CO) groups [6]. Although initially defined by a hydrogen pattern, a secondary structure exhibits a regular geometry that is constrained to a specific region of dihedral angles in the Ramachandran plot [7]. With the exception of the secondary structure assignment program DSSP, which only employs hydrogen bonding information [8], a dozen structure assignments programs using geometric features of local substructures and C*α* atoms have been proposed [3, 9] and programs that employ hydrogen bonds and geometrical restraints have also been applied [7]. Although every program has its benefit, DSSP and STRIDE are the most popular secondary structure assignment programs [7, 8]. Several categories of secondary structures have been noted to occur more frequently in the functional site, especially in the ligand site, including the *π*-helix [10–12], the left-handed helix [13], and the 3_10_-helix in membrane proteins [14], stretches of amino acids with unusual backbone conformations are also frequently observed at ligand-binding sites [15]. These provided insightful heuristics for predicting protein ligand-binding site, but previous research did not explore the correlation between the local amino acid geometric features and ligand-binding site in detail.

Proteins perform their biological functions by binding to other molecules. The binding partner, which is commonly referred to as a ligand, may consist of small organic/inorganic molecules, metals and macromolecules, such as protein or DNA. In this paper, we only consider organic molecules as ligands. The identification of ligand-binding site, especially the primary residues in ligand-binding site, is an important step towards the characterization of their molecular function and rational drug design [16]. Numerous methods have been developed to address this problem; they can be categorized into two groups: sequence-based methods and structure-based methods [17]. Sequence-based methods explore the sequence conservation in proteins under the assumption that ligand-binding site sequences are conversed in the evolution process. Structure-based methods employ geometry criteria to detect a concave region on the surfaces of proteins that forms a surface-solvent-surface event. Due to an increase in the number of known protein ligand complexes in the Protein Data Bank (PDB) [18], some programs utilize known protein ligand structures as templates. Previous studies have revealed that specific backbone conformations are likely to be a part of ligand-binding site and that the magnitude of dihedral angles may undergo slight changes after ligand binding [13, 15, 19]; however, no method has considered the conformation of amino acids in ligand-binding site prediction. Therefore, an extensive survey of the correlations between the value of amino acid dihedral angles and ligand-binding sites was conducted. This information was also employed in the prediction of ligand-binding sites. The discovery of the preference of certain dihedral angle values not only provides a comprehensive overview of amino acid conformation features in protein ligand-binding sites but also facilitates the design of binding site prediction methods.

## 2. Method and Materials

### 2.1. Hydrogen Bond

The hydrogen bonds in a structure were calculated using the program HBPLUS [20]. To identify the hydrogen bonds, this program locates all proximal donor (D) and acceptor (A) pairs that satisfy specified geometrical criteria for hydrogen bond formation. The current criteria are as follows: dist (H-A) < 2.7 Å, dist (D-A) < 3.35 Å, angle (D-H-A) > 90°, angle (H-A-AA) > 90°, where AA is the atom attached to the acceptor.

### 2.2. van der Waals (vdW) Contact

If the distance between the nonhydrogen atom A1 and the nonhydrogen atom A2 satisfies the following criteria,

(1) $$\begin{array}{c} \text{distance}<\text{vdW}\left(\;\text{A1}\;\right)+\text{vdW}\left(\;\text{A2}\;\right)+0.5\;Å, \end{array}$$

where vdW(A*i*) is the van der Waals radius of A*i*, then A1 and A2 are considered to be in vdW contact.

### 2.3. Solvent-Accessible Surface Area (ASA)

The ASA was calculated using the program NACCESS [21]. The default probe size was employed, and any water molecules, hydrogen, or remaining HET groups in the PDB files were disregarded (including the default behaviour). As dihedral angles are determined by the main chain atoms, side chain atoms were not involved in our ASA calculation. The relative accessibility of the main chain of each residue was calculated as the percentage accessibility and was compared with the accessibility of the residue type in an extended ALA-x-ALA tripeptide (for amino acids).

### 2.4. Molecular Potential Energy for Residues

The molecular potential energy for different dihedral angle residues was calculated by the program Open Babel Obenergy (using the AMBER force field) [21]. The positions of the amino acid atoms were directly extracted from the PDB files, and the theoretical hydrogen atom positions of the residues were calculated with the REDUCE program [22]. The equation for calculating the energy for each residue is as follows:

(2) $$\begin{array}{c} E_{total}=E_{bond}+E_{angle}+E_{torsion}+E_{vdW}+E_{electrostatic}, \end{array}$$

where the variables correspond to the bond, angle, torsion, vdW force, and electrostatic force in the mechanical force field, which were evaluated with a nonbonded cut-off.

### 2.5. Three Residue Levels

We classified the binding site residues into three levels. Level 1 residues are strict binding site residues, which are defined as residues that are in direct contact with the ligand, that is, at least one pair of nonhydrogen atoms—an atom from the residue and an atom from the ligand—is positioned with 4 Å distance. If any atom of a residue is positioned at a distance that is less than 6 Å from any atom of the ligand, the residue can be identified as a larger-scale ligand-binding site residue, that is, level 2 residues. Residues are assigned as level 3 residues if the nearest distance between a ligand and residue is less than 15 Å. A binding site secondary structure corresponds to a secondary structure that contains at least one level 2 residue.

### 2.6. Database of Protein Structure

To analyse the protein structures and evaluate the performance of our ligand-binding site prediction method, we downloaded a set of ligand-bound proteins that were determined by X-ray crystallography at a maximum resolution of 2.0 Å; each structure has a maximum identity of 70%. For multichain proteins, the chains share a maximum sequence identity of 30%; otherwise, only one chain is retained. For the selection of ligands, we searched the PDB file for structures with ligands, which are listed in the HETATM (hetero atom) records. We excluded metal ions and inorganic anions, such as Na^+^, Ca^2+^, Cl^−^, PO_4_^3−^, and SO_4_^2−^, from our definition of ligands. Of the 8,189 chain structures, we randomly selected set *T*, which contains 1000 chain structures as our prediction method testing set and defined set *L* as the remaining 7,189 chain structures. The length of the chains in set *L* varies from 22 residues to 1083 residues. To prevent the influence of the geometric size of structures, especially very large complexes, that is, to achieve greater uniformity among the structures, we only retained residues with at least one nonhydrogen atom that is less than 15 Å from the nearest ligand. Nagy and Oostenbrink [4] classified the Ramachandran map into 19 distinct regions on plots based on the observed cluster centre and the density map of (*ϕ*, *ψ*). These regions were used to classify our ligand-prefer *ϕ*/*ψ* boxes.

## 3. Result and Discussion

Left-handed helices and *π*-helices are typical secondary structure types that prefer to stay in the ligand-binding site. Of 31 verified left-handed helices (a minimum of four consecutive residues), Novotoy reported that 27 of the 31 left-handed helices perform an important role either for stability or for the function of the protein [13]. *π*-helices were tended to be associated with a function and ligand-binding site as they were evolutionarily derived from the insertion of a single residue into an *α*-helix [11].

We employed the left-handed helix assignment criteria (*ϕ* of the residues in the left-handed helix fell between 30° and 130°, and *ψ* of the residues lay between −50° and 100°) and the hydrogen bond information calculated by DSSP (version 2.2.1) to detect left-handed helices [13]. SECSTR, a program specifically developed to improve the detection of *π*-helix, was employed to assign *π*-helix in this paper [12].

A total of 6,238 *π*-helix residues (assigned by SECSTR) with at least one atom less than 15 Å from its nearest ligand were detected. The red-edged box (−90° < *ϕ* < −45°, −65° < *ψ* < −37°), which is centred in the *α*1 region defined by the DISICL program, contains 3,263 residues (Figure S1 in Supplementary Material available online at http://dx.doi.org/10.1155/2015/757495). A total of 692 residues (21.2% of 3,263) in the red box were detected at a ligand-binding site compared with 813 (27.3% of 2,975) residues outside the red box, which have at least an atom at a distance less than 6 Å from the ligand. Although the probability for both of these two regions reside at a ligand-binding site exceeds the average level (19.8%), the difference between them is significant (21.2% compared with 27.3%). After searching all structures in the set *L*, 88.2% of the residues in the region in the red-edged box were assigned as *α*-helices. Although the *α*-helix is the most common secondary structure, existing data have not determined a correlation with protein functions. The divergence suggests that *π*-helix residues have variant preferences at binding sites with different backbone dihedral angles.

**Figure 1:**
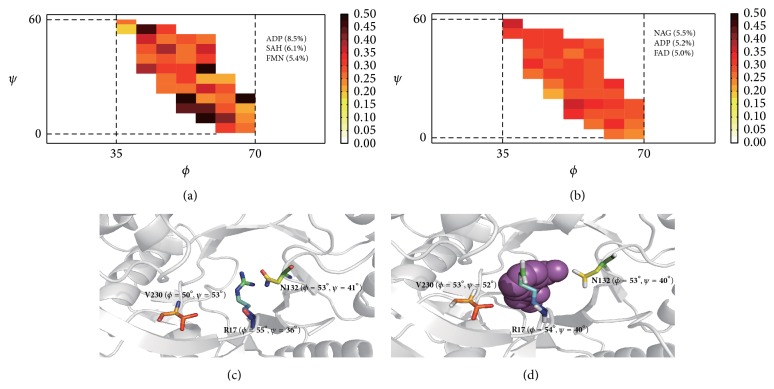
Probability for (a) left-handed helix residues and (b) non-left-handed helix residues observed at ligand-binding site. (a) shows the probability of left-handed helix residues being observed in a ligand-binding site; (b) illustrates the probability of non-left-handed helix residues observed at a ligand-binding site in the same region. The top three most frequent ligands contacted with left-handed helix residues (a) and non-left-handed helix residues (b) are also labelled as three-letter code in (a) and (b). (c) shows an example of non-left-handed helix residues at a ligand-binding site. In (a) and (b), the probability value, which is expressed as a percentage, is defined by the number of residues detected in the ligand-binding site divided by the total number of residues observed in the 5° × 5° Ramachandran box. (c) and (d) show examples of non-left-handed helix residues (coloured residues) at a ligand-binding site in O-succinylbenzoate synthase with a ligand-free form in (c) (pdbid: 2opj) and a ligand-bound form in (d) (pdbid: 2qvh). The dihedral angles for the residues are noted in bold font, and the ligand is indicated by purple spheres.

A comparison between left-handed helix residues and non-left-handed helix residues in the same *ϕ*/*ψ* region as left-handed helix residues is provided. First, we determined the probability for left-handed helix residues in different *ϕ*/*ψ* boxes observed at the ligand-binding site, which are denoted as coloured boxes in Figure 1; boxes with fewer than five residues were excluded. Second, we calculated the probability for non-left-handed residues with dihedral angles in the same region as left-handed helix residues in the Ramachandran plot, which are denoted by the identically coloured boxes observed in the ligand-binding site (Figure 1(b)). Of the 1,328 left-handed helix residues detected in set *L*, 426 were located at a ligand-binding site compared with 4,557 out of the total of 15,263 non-left-handed helix residues (78% of the non-left-handed helix residues were assigned as “Turn” or “Bend” by DSSP). A higher probability at the ligand-binding site was observed for the left-handed helix residues (32.1% compared with 29.8%) because a left-handed helix requires two consecutive amino acid dihedral angles that are positioned in the coloured region. Figure 1 shows the detailed probabilities for left-handed helix and non-left-handed helix residues in the same region.

The examples of *π*-helices and left-handed helices suggest that residues in a *π*-helix exhibit different performances, which correlate with a ligand as their dihedral angle changes. The residues in a specified region (coloured boxes in Figure 1) yield similar probabilities of detection at a ligand-binding site. These findings inspired us to search the database to determine whether other ligand-prefer Ramachandran regions exist, such as left-handed helix dihedral angle regions, which have a preference for protein ligand-binding sites, instead of focusing on the secondary structure level, as noted in previous studies [10–14].

**Figure 2:**
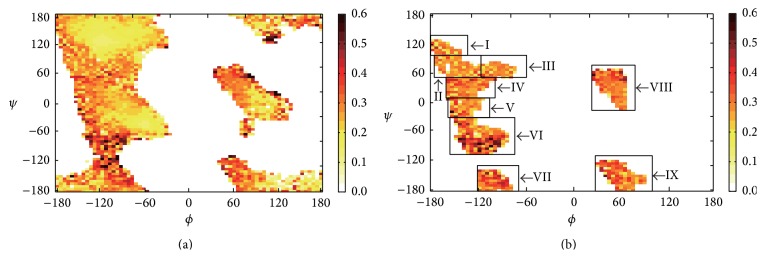
Observed probabilities at ligand-binding site for (a) 5° × 5° Ramachandran boxes and (b) ligand-prefer Ramachandran boxes in nine regions. The probability increases from white to yellow to orange to black; the boxes in both figures with probabilities > 0.6 are also represented as black boxes. Angles are shown in degrees. Detailed *ϕ*/*ψ* boundaries for the nine regions are shown in Table S1.

We employ the probability *P*_*ϕ*,*ψ*_ as a measure for a 5° × 5° Ramachandran box that is observed at a ligand-binding site. *P*_*ϕ*,*ψ*_ is calculated by the number of level 2 residues divided by the total number of level 3 residues, and the dihedral angles of both of these level 2 residues are located in the 5° × 5° Ramachandran box. Boxes that consist of less than 20 level 3 residues were excluded. A total of 972,773 level 3 residues, of which 192,606 are level 2 binding site residues, with an average probability for residues of 19.8%, were detected at a ligand-binding site. The probability *P*_*ϕ*,*ψ*_ for every 5° × 5° Ramachandran box is shown in Figure 2(a).

**Figure 3:**
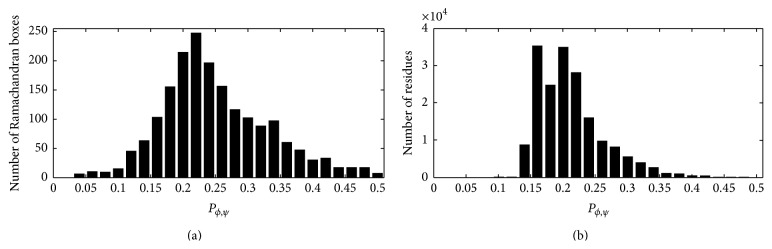
Observed distribution for (a) 5° × 5° Ramachandran boxes and (b) level 2 binding site residues. The *x* axis for both figures indicates the probability observed at the ligand-binding site; the number is labelled on the *y*-axis.

To prevent random occurrence of Ramachandran boxes that have high *P*_*ϕ*,*ψ*_ values themselves but low *P*_*ϕ*,*ψ*_ neighbours, we also considered the neighbours of the boxes. The top 35% of boxes according to *P*_*ϕ*,*ψ*_ value (with *P*_*ϕ*,*ψ*_ > 28%) were selected as central high *P*_*ϕ*,*ψ*_ value boxes, and the neighbours' average *P*_*ϕ*,*ψ*_ values must be in the top 45% in terms of *P*_*ϕ*,*ψ*_ value for all boxes. Thus, ligand-prefer Ramachandran boxes are defined as follows: if the Ramachandran box *P*_*ϕ*,*ψ*_ > 28% and the average probability for the four neighbouring boxes (up, down, left, and right boxes) exceeds 26%, the five boxes are defined as ligand-prefer Ramachandran boxes. Combined with Ramachandran regions, DISICL has defined our ligand-prefer Ramachandran boxes as distributed among nine Ramachandran regions (Figure 2(b)). Among all 1884 boxes in Figure 2(a), 827 (43.3%) boxes have a probability greater than 26%, whereas level 2 binding site residues in these 827 boxes comprise 12.4% of all level 2 residues. The distributions of the number of boxes and the level 2 binding site residues for different probability levels are shown in Figure 3.

When examining the composition of the level 2 residues in nine ligand-prefer *ϕ*/*ψ* regions, the total number of level 2 residues in ligand-prefer Ramachandran region VIII is twice as large or more in terms of the other eight regions (Table 1). Asp occurs most frequently in regions I, II, III, and IV, with a minimum probability of 35% in the ligand-binding site in these four regions. Level 2 His demonstrates the second, third, third, and second largest contributions to region I to IV; its propensity at the ligand-binding site is always in the top three in all nine regions. Gly is notable because it accounts for 25% of regions VII and VIII and 72.1% of region IX. Cys has the largest probability detected at a ligand-binding site from regions VI to IX; however, the number of Cys from regions VII to IX is relatively low. Ala, Lys, and Pro have relatively low propensities for ligand-binding site occurrence, with the exception of Ala in region IX with a probability of 37.3% at the ligand-binding site. The secondary structure assigned by DISICL suggests that the secondary structures for the ligand-prefer *ϕ*/*ψ* boxes are promiscuous and do not show a preference for specific secondary structures. We define the probability value *P*(AA, *r*) as a measure of propensity at a ligand-binding site, where AA is the amino acid and *r* is the ligand-prefer Ramachandran region index; *P*(AA, *r*) will be employed in our ligand-binding site prediction scoring function. Figure S2 shows the distribution of 20 amino acids in each region and the probabilities observed at ligand-binding sites. For statistical analysis, ligand-preference of different regions is compared using a two-tailed Wilcoxon Rank-Sum test; the *p* values for ligand-preference for any two ligand-prefer Ramachandran regions are available in Table S3. Nine ligand-prefer Ramachandran regions all demonstrate significant difference with the other region (the region except for the nine regions in the Ramachandran plot); however, only a few *p* values are less than 0.05 within any two of the nine regions

(3) $$\begin{array}{c} P\left(\;AA,r\;\right)=\frac{\text{total number of level 2}\;\;AA\text{ in region }r}{\text{total number of level 3 }AA\text{ in region }r}. \end{array}$$

### 3.1. Molecular Potential Energy

In 1991, Herzberg reported that sterically strained (*ϕ*, *ψ*) residues are energetically unfavourable by calculating the energy for N-acetyl-N′-methylalanyl amide with geometry optimization of bonds, bond angles, and torsions [23]. A detailed energy comparison was also performed for ligand-prefer region residues in each region, and the average level was calculated using all residues from set *T*. The program Open Babel Obenergy was employed for the energy calculations [24]. The exploration of a specific amino acid in different regions with slight variations in energy is insignificant because the potential energy function is empirical and several limitations produce inaccuracies in the calculated potential energy. As shown in Table 2, residues in the nine regions have similar or slightly higher potential energy compared with the average level, with several exceptions. Ala, Gly, Ser, His, and Lys have molecular potential energy within 10 KJ/mol compared with the average level, which shows that these residues have low divergence in energy in different regions. The highest molecular energy for Val, Leu, Phe, Gly, and Asp is observed in region IV, whereas the highest energy for Met, Try, His, Glu, and Asn is observed in region VII. Region IX consists of the four highest energy amino acids: Cys, Ser, Gln, and Arg. The energy increases by 45.9 KJ/mol in region VII and 32.2 KJ/mol in region IX compared with the average level for Met.

### 3.2. Solvent-Accessible Surface Area (ASA)

**Figure 4:**
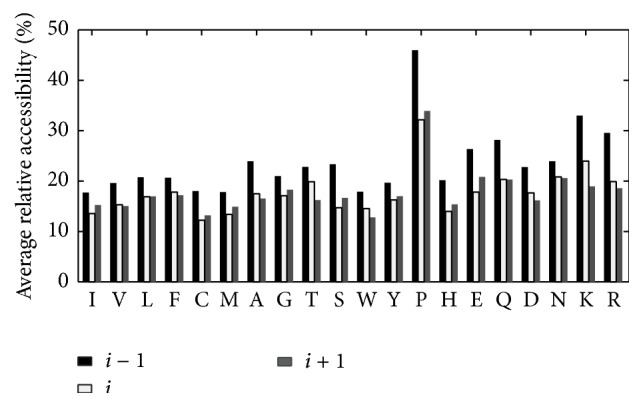
Average relative accessibility for ligand-prefer boxes residues (*i*) and their neighbours (at positions *i* − 1 and *i* + 1).

The two dihedral angles are determined by the backbone atoms of proteins. To understand solvent-accessible areas for ligand-prefer box residues and their neighbours, we calculated the relative accessibility for the backbone of ligand-prefer box residues (*i*) and their neighbours (*i* − 1, *i* + 1). As depicted in Figure 4, the ASA for the (*i* − 1) amino acid has a significantly higher relative accessibility surface compared with the remaining two positions, which indicates that ligand-prefer box residues are buried more and their previous residues are exposed more to solvent.

### 3.3. Hydrogen Bonds and vdW Contacts

**Figure 5:**
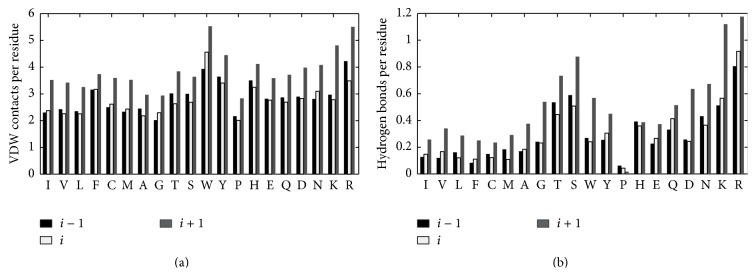
Number of residue-ligand VDW contacts (a) and residue-ligand hydrogen bonds (b) for ligand-prefer Ramachandran boxes residues (*i*) and their neighbours at positions *i* + 1 and *i* − 1.

Hydrogen bonds and vdW contacts are the two main noncovalent contacts between ligands and amino acids.  Figure 5 shows the average vdW contacts and hydrogen bonds formed by residues (*i* − 1, *i*, *i* + 1) and ligands; ligand-prefer *ϕ*/*ψ* box residues are at position *i*. All amino acids have the most number of vdW contacts with a ligand at position *i* + 1, with an additional 1.5 and 1.6 contacts/residue for Asp at position *i* + 1 compared to position *i* − 1 and position *i*. For the amino acids in positions *i* and *i* − 1, the number of vdW contacts with ligands is similar, with the exception of Trp and Arg. Residues at position *i* + 1 have more than 0.8 vdW contacts/residue with ligand than the other two positions, with Ile, Val, Met, Asp and Arg, and Lys having 1.4 more vdW contacts/residue with ligand. Obviously, residues at position *i* + 1 are capable of providing more contacts with ligand than positions *i* and *i* − 1.

Figure 5(b) delineates the average number of hydrogen bonds established by ligand and residues at three positions as mentioned above. Almost all residues at position *i* + 1 formed more hydrogen bonds/residue with ligand, while only Pro at position *i* − 1 established the greatest number of hydrogen bonds/residue with compound. Two irregular amino acids are Pro and His, with fewer hydrogen bonds at position *i* + 1. Half of the 20 amino acids at position *i* + 1 have twice or more hydrogen bonds/residue with ligand than the other two positions.

In contrast with our previous assumptions, these ligand-prefer box residues do not achieve greater direct interaction with ligands, and the special geometry conformation results in the amino acids following ligand-prefer box residues forming more hydrogen bonds and vdW contacts with ligands.

### 3.4. Prediction of Protein Ligand-Binding Sites Based on Dihedral Angles

We demonstrated the ability of dihedral angle-based prediction, as previously discussed, in the context of blind prediction and the Ligsite-csc program [25]. The prediction comparisons were made using the set *T*, which consists of 1000 protein structures with a maximum identity of 70%. If any two chains in a protein had an identity greater than 70%, we only employed the first occurrence chain in the PDB file. Ligands that bind to other chains were excluded.

The prediction procedure followed a sequence of three steps. First, we employed the Ligsite-csc program to locate solvent grids in a protein. Ligsite-csc calculates all grids that encompass the protein structure. These grids are divided into three categories: protein grids, surface grids, and solvent grids. A protein grid has at least one protein atom within 1.6 Å. Surface grids have a Connolly vertex within 1.0 Å, and all other grids are characterized as solvent grids [25]. Our method and Ligsite-csc only used the solvent grid points in ligand-binding site prediction. Second, we assigned the score *h*_*i*_ to each grid point *i*:

(4) $$\begin{array}{c} h_{i}=1-\overset{n}{\underset{j=1}{\prod }}\left(\;1-P\left(\;AA_{j},r\;\right)\;\right), \end{array}$$

where *P*(AA, *r*) is defined in (3) and *n*  (*n* > 1) is the total number of ligand-prefer Ramachandran box residues that are positioned less than 6 Å from the grid point *i*. Last, we sorted the grid points in descending order based on the scoring *h*_*i*_. The prediction results corresponded to the top-scoring grid point.

The predictions were evaluated based on the distance between the top-scoring grid and the actual position of the ligand; that is, a prediction was assumed to be correct if the distance was less than the cut-off threshold value, which varies from 1 to 10 Å. For a given protein structure, we only considered the top-scoring grid point. If the values of any atoms in the ligand were less than the cut-off threshold value from the point, the prediction was assumed to be correct. The success rate was defined as the number of correctly predicted proteins divided by the total number of dataset structures.

Our method was compared with Ligsite-csc (an extension of Ligsite), which identifies pockets based on the notion of surface-solvent-surface events and the degree of conservation of the involved surface residues [25]. Ligsite-csc performs slightly better than other predictors, such as Ligsite, CAST, PASS, and SURFNET [25]. We also implemented a baseline predictor by randomly selecting a grid point that was indicated as a solvent grid by Ligsite-csc.

We also created a set of 362 structurally distinct ligand-free proteins that share more than 95% structural similarity with the ligand-bound form. This was achieved by examination of the ligand-bound-free pairs from the Comsin database [26]. The ligand-bound complexes were superimposed onto their corresponding ligand-free proteins. The ligand coordinates were extracted for ligand-free structure ligand-binding site prediction.

**Figure 6:**
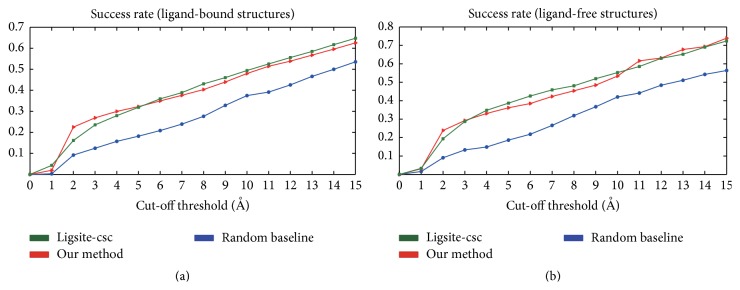
Performance of ligand-binding site prediction, including Ligsite-csc, our method, and a random baseline predictor for ligand-bound structures (a) and ligand-free structures (b). The *y*-axis represents the success rate, that is, the nearest distance between the predicted binding site and any atom of a ligand, which is less than or equal to the distance labelled on the *x*-axis.

**Figure 7:**
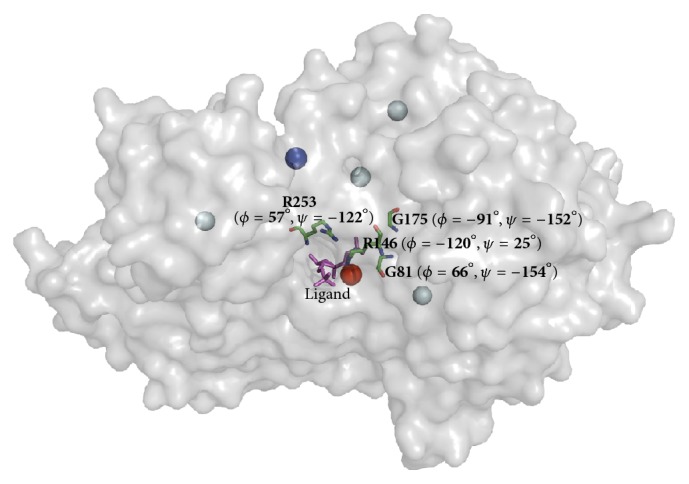
An example of the ligand site prediction performance of Ligsite-csc and our method (pdbid: 2f48). The top-scoring binding site predicted by Ligsite-csc is denoted by a blue sphere, four additional binding sites listed in top five score grids predicted by Ligsite-csc are denoted by light blue spheres, and the site predicted by our method is denoted by a red sphere. The protein surface is depicted in grey, and the ligand is shown as a purple stick. The *ϕ*/*ψ* angles for four ligand-prefer Ramachandran boxes residues around the red sphere are indicated in bold font.

Gunasekaran and Nussinov reported that the magnitude of the dihedral angle changes is minimal after ligand binding [19], which explains why our method has a performance similar to that of Ligsite-csc for both ligand-bound and ligand-free protein ligand-binding site prediction when only one potential pocket is predicted, as shown in Figure 6. The success rate of our method is even higher than that of Ligsite-csc when the distance threshold value is set to 2 Å; however, the success rate of our method increases at a slower rate than that of Ligsite-csc when the distance increases. Our method and Ligsite-csc are both superior to random selection. A total of 82 ligand-binding sites (cut-off threshold = 4 Å) were successfully predicted by our method but could not be detected by Ligsite-csc even when three potential pocket sites were predicted (Table S4). An example is shown in Figure 7; the top prediction by Ligsite-csc is 13 Å from the ligand, and the distance between our prediction grid and the compound is only 1.7 Å. For the remaining top five grids predicted by Ligsite-csc, the shortest distance between the grids and the small molecule was 18 Å. We also provided a comparison for a particular binding site: “HEM” (protoporphyrin IX containing Fe) binding site (Table S5). The results indicate that our method is a useful tool for ligand-binding site prediction, especially for predicting a site that is less than 2 Å from a ligand.

## 4. Conclusions

We have enumerated the ligand-prefer Ramachandran boxes, for which residues have a high probability of being observed in the ligand-binding site, and classified these boxes into nine regions. Instead of direct contact with ligands, residues preceding ligand-prefer Ramachandran boxes are exposed more to solvent compared with the residues following ligand-prefer Ramachandran boxes, which form more vdW contacts and hydrogen bonds with ligands. This pattern suggests that residues in ligand-prefer Ramachandran boxes and their preceding amino acids facilitate subsequent residue contact with ligands. Residues in ligand-prefer Ramachandran boxes are irregular; common secondary elements for these residues are “undefined” as assigned by DISICL. The relative propensity observed at ligands for residues with specific *ϕ*/*ψ* values should aid in the identification of binding sites in proteins.

Our score function in ligand-binding site prediction is based on the propensities of amino acids. Typically, Cys is heavily weighted when its *ϕ*/*ψ* angle is located in a region V Ramachandran ligand-prefer boxes due to its high propensity (73.2%). Cys has a high propensity in all nine ligand-prefer Ramachandran regions, varying from 41.8% to 69.7%. Several algorithms have been published for predicting ligand-binding sites, and critical information, such as information about geometry, amino acid composition, physical potential, and ligand-binding residues that are conserved in the evolutionary process, has been employed for predictions. We first demonstrate a practical application using the residue *ϕ*/*ψ* angle in the context of blind prediction and the program Ligsite-csc. Our analysis reveals that a scanning method based on the simple propensity of the *ϕ*/*ψ* angle performs as well as Ligsite-csc when one ligand-binding site is predicted. The use of the *ϕ*/*ψ* angle to predict ligand-binding sites can be a useful tool for various aspects of drug discovery.

## Supplementary Material

Supplementary material consists of five tables, and two figures: Figure S1 shows the distribution of π-helix residue dihedral angles while the distribution of 20 amino acids in each region and the probabilities observed at ligand-binding sites is shown in Figure S2. Table S1 illustrates the ϕ/ψ boundaries for the nine regions and Table S2 describes abbreviation codes for secondary structures assigned by DISISL. P-values of Wilcoxon Rank-Sum test for the ligand-preferences on the ten Ramachandran regions are shown in Table S3. Table S4 lists 82 ligand-binding sites that Ligsite-csc fails to detect but our method successfully predicts, and Table S5 displays a comparison of Ligsite-csc and our method at the “HEM” binding site.

## Figures and Tables

**Table 1:** Distribution of amino acids in nine ligand-prefer Ramachandran regions.

Region	Number 1^1^	Number 2^2^	Probability^3^	Top three AA occurrences^4^	Top three AA observed at ligand site^5^	Secondary structure features^6^	Top three frequently ligand^7^
I	1,525	5,366	0.284	D(207), H(138), S(132)	H(45.8%), E(38.5%), G(36.3%)	EBS(24.7%), NBS(23%), HC(17.2%)	NAG(4.9%), FAD(3.8%), FMN(3.4%)

II	1,269	4,262	0.297	D(150), N(140), H(123)	H(41.8%), E(41.2%), W(39.0%)	TC(18.2%)	FAD(6.8%), NAG(4.6%), HEM(4.2%)

III	1,895	6,779	0.279	D(329), N(218), H(153)	H(53.7%), G(42.3%), M(36.1%)	GXT(40.1%)	FAD(6.4%), HEM(4.3%), HAG(3.3%)

IV	2,693	9,373	0.287	D(327), H(224), N(295)	H(45.6%), D(39.5%), A(37.7%)	TC(16.8%), SCH(11.9%), BU(9.1%)	FAD(8.4%), NAG(5.8%), HEC(3.5)

V	2,164	7,815	0.276	T(258), D(157), V(141)	C(73.2%), H(43.5%), D(39.6%)	BU(20.7%), SCH(15.1%), TC(14.4%)	HEM(5.8%), FAD(4.1%), NAG(3.7%)

VI	2,369	7,714	0.307	V(247), I(232), L(217)	C(63.3%), H(44.7%), W(33.1%)	*α*H(13.9%), *π*H(12.5%), BU(7.1%)	HEM(6.4%), FAD(4.7%), FMN(3.0%)

VII	1,690	5,208	0.325	G(837), D(207), N(113)	C(67.3%), H(51.2%), R(41.8%)	HC(24.0%), PP(12.5%), BC(7.6%)	FAD(9.5%), NAD(4.8%), NAG(4.6%)

VIII	5,974	20,007	0.298	G(1171), N(840), D(695)	C(67.1%), H(51.3%), R(41.8%)	SCH(31.5%), LHH(17.8%), TC(15.9%)	NAG(7.5%), FAD(3.4%), SAH(3.3%)

IX	1,482	4,609	0.322	G(1067), S(101), A(46)	C(72.2%), H(69.7%), S(59.4%)	LT2(51.1%)	FAD(9.2%), SAH(6.6%), SAM(4.1%)

Other^8^	171,545	901,640	0.190	L(90,807), A(72,088), V(69,604)	H(29.2%), C(27.6%), W(27.2%)	ALH(28.0%), EBS(12.9%), HC(9.5%)	FAD(5.4%), HEM(4.9%), NAG(3.0%)

^1^Total number of level 2 residues in region *n* ligand-prefer Ramachandran boxes, where *n* ranges from I to IX.

^2^Total number of level 3 residues in region *n* ligand-prefer Ramachandran boxes, where *n* ranges from I to IX.

^3^The value in this column is calculated by the number in column 2 divided by the number in column 3.

^4^The top three occurrences level 2 residues in region *n* ligand-prefer Ramachandran boxes.

^5^Probability is calculated by the number of level 2 residues in region *n* ligand-prefer Ramachandran boxes divided by number of level 3 residues in the region *n* ligand-prefer Ramachandran boxes.

^6^Only residues that are not assigned as “undefined” are listed; for additional information about DISISL assignment, refer to Table S2.

^7^Top three most frequent ligands (three-letter code in PDB file) contacted with level 2 residues in the region.

^8^The other is the remaining region (except the nine regions) in the Ramachandran plot.

**Table 2:** Molecular potential energy for 20 amino acids in nine ligand-prefer Ramachandran regions (values in KJ/mol).

AA	Average^1^	I	II	III	IV	V	VI	VII	VIII	IX
I	69.8	72.1	84.7	84.4	69.8	82.4	77.6	64.8	71.4	78.4
V	47.4	45.5	59.0	58.2	**72.2** ^2^	**63.0**	55.7	**69.7**	**61.7**	**62.6**
L	70.6	73.9	74.7	71.9	**87.6**	69.7	73.8	82.1	73.2	79.1
F	80.0	91.2	90.3	90.6	**99.7**	**98.1**	91.7	90.0	82.0	81.3
C	38.0	37.7	42.2	42.3	43.6	43.9	**46.5**	42.9	40.4	**56.9**
M	59	63.5	55.4	67.2	71.7	70.0	67.5	**104.9**	61.8	**91.2**
A	31.9	31.7	37.6	33.4	31.9	38.6	30.7	32.8	33.4	29.4
G	27.2	24.3	30.6	30.9	36.9	35.9	37.2	31.4	34.1	31.3
T	49.7	44.5	51.3	53.3	54.5	46.4	51.2	56.0	**69.1**	47.5
S	42.6	44.4	44.3	45.5	48.5	47.7	47.4	45.6	41.8	50.3
W	203.7	203.3	208.7	208.3	211.4	212.8	210.5	214.2	206.5	211.9
Y	82.1	92.9	82.3	82.1	91.9	91.3	87.7	87.5	87.5	84.5
P	109.5	—	—	—	115.2	109.9	—	117.4	—	—
H	193.4	187.6	193.2	190.4	192.8	190.0	193.5	200.4	193.1	190.7
E	68.2	60.5	73.1	73.4	64.3	69.5	70.6	79.8	66.0	52.5
Q	36.6	42.4	46.7	47.4	37.6	**56.2**	37.2	40.9	38.7	**66.9**
D	71.1	67.8	74.8	71.7	85.1	76.4	72.0	84.5	73.2	73.0
N	41.8	35.2	46.8	42.1	52.2	46.3	39.0	**84.4**	45.8	43.7
K	74.6	80.5	76.8	76.2	74.0	79.3	79.4	71.5	71.9	62.4
R	208.6	206.8	207.9	204.4	208.9	209.7	209.1	209.5	208.1	**236.9**

^1^Average energy is calculated by residues that are not in the nine ligand-prefer Ramachandran regions; outliers energy calculation (*E* > 1000 KJ/mol) are excluded.

^2^Energy values that are 15 KJ/mol higher than the second column are denoted in bold.

“—” represents regions in which Pro does not occur.
