# Supplementary material for: Amino Acids in Nine Ligand-Prefer Ramachandran Regions
Source: Biomed Res Int. 2015 Sep 29;2015:757495. doi: 10.1155/2015/757495 (PMC4602322; doi:10.1155/2015/757495)
Supplement: Supplementary file 1 — Supplementary material consists of five tables, and two figures: Figure S1 shows the distribution of π-helix residue dihedral angles while the distribution of 20 amino acids in each region and the probabilities observed at ligand-binding sites is shown in Figure S2. Table S1 illustrates the ϕ/ψ boundaries for the nine regions and Table S2 describes abbreviation codes for secondary structures assigned by DISISL. P-values of Wilcoxon Rank-Sum test for the ligand-preferences on the ten Ramachandran regions are shown in Table S3. Table S4 lists 82 ligand-binding sites that Ligsite-csc fails to detect but our method successfully predicts, and Table S5 displays a comparison of Ligsite-csc and our method at the “HEM” binding site. [file 757495.f1.pdf]

Supporting Information:

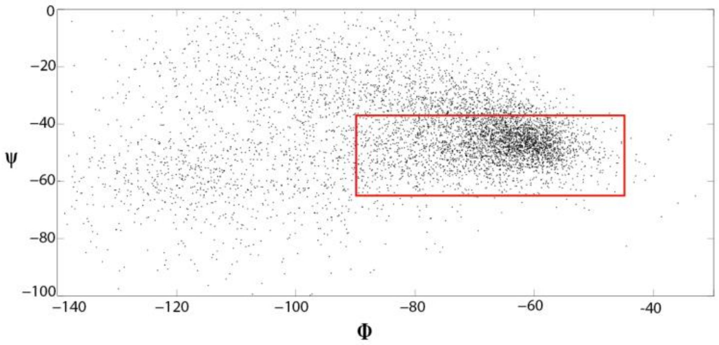

Fig S1: Distribution of π helix residue dihedral angles. The red region inside the rectangle defined by two intervals, [-90°,-45°] for φ and [-50°,100°] for ψ, includes 52.3% of all the 6,238π-helix residues.

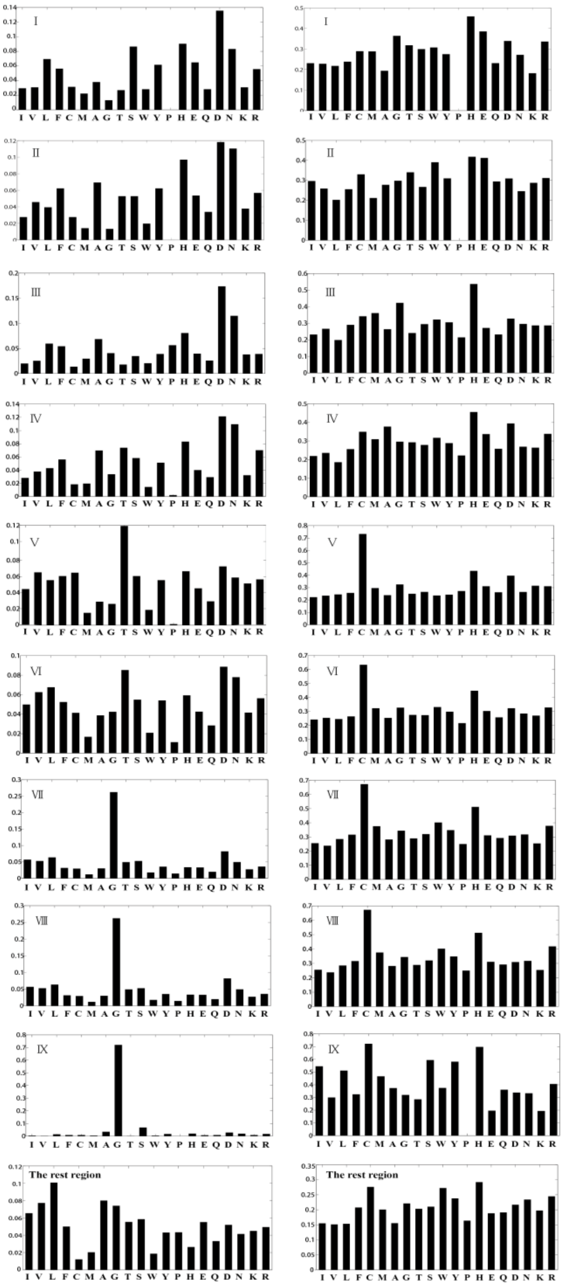

Fig S2: Detailed distribution for 20 amino acids. Distribution of 20 amino acids in nine ligand-prefer Ramachandran regions and the rest region in the Ramachandran plot (ten left figures). The probabilities for 20 amino acids in the region observed at the ligand-binding site are shown to the right.

region	ϕ min	ϕ max	ψ min	ψ max
I	-180	-135	95	136
II	-177	-118	50	95
III	-118	-60	50	95
IV	-162	-100	8	50
V	-160	-107	-32	8
VI	-157	-75	-107	-32
VII	-122	-70	-180	-130
VIII	23	78	-17	75
IX	27	100	-180	-110

Table S1: φ/ψ boundaries for the nine regions. Angles are shown in degrees.

Secondary structure	Abbreviation code
**3/10-helix**	3H
**turn type 1**	T1
**turn-cap**	TC
**α-helix**	αH
**π-helix**	EβS
**helix-cap**	HC
**ext. β-strand**	EβS
**normal β-strand**	NβS
**β-cap**	BC
**PP helical**	PP
**β bulge**	BU
**turn type 2**	T2
**turn type 8**	T8
**γ turns**	GXT
**Schellman turn**	SCH
**hairpin 2:2**	HP
**left turn 2**	LT2
**left-handed helix**	LHH

Table S2: Abbreviation codes for secondary structures assigned by DISISL[4].

	II	III	IV	V	VI	VII	VIII	IX	Other[1]
I	0.399	0.407	0.374	0.535	0.373	**0.043**	**0.041**	**0.005**	**0.002**
II		0.660	0.661	0.361	0.752	0.198	0.181	**0.011**	**7.406e-05**
III			0.703	0.715	0.681	0.136	0.134	**0.013**	**4.166e-05**
IV				0.618	0.703	0.208	0.190	**0.015**	**3.705e-05**
V					0.351	**0.0483**	**0.0448**	**0.012**	**2.596e-05**
VI						0.172	0.113	**0.011**	**7.572e-06**
VII							0.878	0.057	**6.907e-07**
VIII								0.082	**6.917e-07**
IX									**3.712e-05**

Table S3: Wilcoxon Rank-Sum test for the ligand-preferences of the ten Ramachandran regions. The matrix contains the Wilcoxon Rank-Sum p-values for a 2-tailed test for the ligand-preference of any two regions. Ten regions include the nine ligand-prefer Ramachandran regions mentioned in the paper and the rest region (last column) in the Ramachandran plot. Significant p-values ($p < 0.05$) are labelled in bold. P-values were calculated using the MATLAB “ranksum” function.

[1]The other region is the rest region (except the nine regions) in the Ramachandran plot.

pdbid	Ligand	h_score[1]	Our_distance[2]	Lig-csc_distance[3]	Residue_number[4]
**1e6u**	NAP	0.53438	1.65965	8.49423	106 164
**1g8k**	MGD	0.680314	1.36526	9.93683	166 196 456
**1h1o**	HEM	0.865643	1.18059	10.9398	15 16 19 64
**1i2k**	PLP	0.58835	3.47231	7.13356	140 144 176
**1jg1**	SAH	0.519445	1.44526	8.24954	72 101 165
**1kkh**	DIO	0.570153	1.75772	5.44562	111 113 115
**1kqf**	MGD	0.801787	1.45544	15.4063	556 558 902 904
**1m15**	NO3	0.547144	3.38134	8.45093	114 117
**1n97**	HEM	0.62756	1.64582	5.29361	98 222 223
**1olt**	SF4	0.550465	1.10811	5.06743	113 184
**1r6w**	164	0.612506	1.82502	4.66365	288 289 290 291
**1su8**	NFS	0.909122	1.16605	9.97441	261 294 333
**1vrm**	MRD	0.643478	1.75609	7.47402	164 183 184
**1xku**	NAG	0.547289	2.77492	4.35566	154 180 181 201 203
**1zel**	MPD	0.55791	1.32015	20.6569	172 173
**2a9d**	MTE	0.578506	1.61581	10.548	299 302
**2bkx**	F6R	0.554315	1.28463	10.9081	36 37 132 140
**2ce7**	ADP	0.591875	3.9393	4.84879	165 166
**2e2e**	IMD	0.667506	2.8315	4.96436	158 203
**2f48**	FBP	0.610721	1.9939	13.1438	81 146 175 253
**2g8l**	UNL	0.65035	2.86658	4.22164	188 189
**2gks**	ADP	0.763661	2.47544	5.4	382 450
**2i5e**	TRS	0.570153	2.57795	4.24022	120 121 122 140 141
**2iw1**	U2F	0.547658	1.6508	9.18236	98 143 281
**2jae**	FAD	0.554315	1.64904	9.67098	21 49 422
**2jhf**	DMS	0.596363	0.473914	4.52501	67 141
**2nzl**	GLV	0.695769	1.10474	12.8192	82 260 263
**2olr**	ATP	0.720468	1.34789	9.42819	232 268 286
**2pzm**	NAD	0.518586	1.74196	4.11745	7 75
**2rgo**	FAD	0.621754	1.09044	18.3409	26 27 231 233
**2ww5**	CHT	0.593787	1.37497	9.90964	84 135
**2xts**	MTE	0.662405	1.60351	7.24921	116 219 263 266
**2yqz**	SAM	0.668535	1.48209	5.85568	49 112
**2zpa**	ACO	0.599221	1.27963	4.11255	469 470
**2zyr**	1PE	0.733893	1.0723	13.4946	32 33 136 426
**3ano**	PG4	0.438109	3.70064	13.3547	85 148

**3bdl**	CIT	0.629044	1.32218	5.31995	393 450
**3c2u**	B3P	0.596646	1.68519	13.0227	14 31 73 508
**3ch0**	CIT	0.599221	1.73975	9.09593	14 137 197
**3d1c**	FAD	0.576837	1.78856	4.19343	137 139
**3dk9**	FAD	0.621754	1.53451	5.15002	28 29 56 157
**3edf**	CE6	0.600888	1.46731	10.2012	418 470
**3fnc**	MLI	0.532131	1.38775	4.10519	95 96
**3gfa**	FMN	0.800874	3.78572	6.25503	150 170
**3hfw**	ADP	0.694061	3.75855	23.2859	165 219
**3i3l**	FAD	0.591875	1.22714	17.9013	12 163 165
**3mqd**	3MQ	0.694271	1.85838	4.51656	160 335
**3o26**	NDP	0.554315	1.58184	6.75326	19 23
**3ooi**	SAM	0.550465	2.03148	6.12876	100 101 102 220
**3p9z**	MLI	0.493039	1.54193	7.25612	119 121
**3qc0**	PG4	0.604306	1.80633	4.95652	105 109 150
**3r0v**	MLT	0.531581	2.33468	5.22935	28 30 156
**3rha**	FDA	0.591875	1.21941	14.6181	15 16 44 264
**3s1x**	I22	0.504674	1.29392	4.87774	108 167
**3tos**	SAH	0.640582	1.66739	10.8171	79 130 189
**3u81**	SAH	0.704836	1.46763	7.58774	41 90 141 142
**3v97**	SAH	0.713765	1.5026	8.06306	546 548
**3wh2**	FLC	0.466593	1.7499	17.4495	171 172
**4a0p**	NAG	0.588213	1.39967	23.4761	673 674
**4c1u**	AHR	0.566439	1.36972	6.81742	146 254
**4c4a**	SAH	0.63858	1.62685	7.99862	74 94 144 153
**4ehu**	ANP	0.570153	1.45393	7.15394	9 11 101 103
**4gbm**	A3P	0.603059	1.46028	11.1563	39 268
**4ido**	GDP	0.618572	1.55404	19.4384	77 78 114
**4iu6**	FZ1	0.68228	1.87294	4.79981	196 212 353
**4jn7**	LMR	0.695769	1.21106	5.73831	191 295
**4k8w**	EOH	0.446871	1.97818	11.0036	79 147
**4lhs**	PEG	0.668535	1.73985	11.9161	326 327 329
**4lii**	FAD	0.621754	1.36588	8.89689	139 140 261 437
**4m24**	CBI	0.610632	1.78393	4.46544	92 135 171
**4mob**	COA	0.467858	2.61528	4.06411	54 114
**4nec**	SAH	0.554315	1.35139	6.34305	47 49 113
**4ntc**	FAD	0.6125	1.65735	7.61529	19 21 43 121 123
**4ob7**	MPD	0.719738	1.93573	7.68399	159 287 290
**4p8n**	R57	0.66162	1.25893	5.60875	315 325
**4pxy**	MES	0.702867	2.11354	15.4191	130 131
**4qhb**	PEG	0.552671	2.85389	7.17509	66 67 252
**4r38**	RBF	0.5244	1.90674	4.36903	29 30
**4r9f**	BMA	0.503521	3.24155	7.90799	190 251
**4tqg**	NDP	0.554303	1.54904	4.01681	129 169
**4u5i**	BXP	0.674034	1.09498	5.93739	93 103 169
**4utu**	LRY	0.53701	1.35625	7.5708	203 208

Table S4: **Comparison of Ligsite-csc and our method.** The table consists of 82 ligand-binding sites that Ligsite-csc fails to detect but our method successfully predicts when the cut-off threshold is set to 4.0 Å.

[1]Top score of our method.

[2]The nearest distance between the top score grid assigned by our method and the ligand.

[3]The nearest distance between the top three score grids assigned by Ligsite-csc and the ligand.

[4]Residue number of ligand-prefer Ramachandran box residues that are positioned less than 6 Å from the top h_score grid.

pdbid	h_score[1]	Our_distance[2]	Lig-csc_distance[3]	Residue_number[4]
1ash	0.604836	4.48077	0.650098	99 100 101
1dw0	0.758116	1.70795	1.016686	40 46 92
1gwe	0.614668	20.2986	18.2057	125 368
**1h1o**	**0.865643**	**1.18059**	**13.6849**	**15 16 19 64**
1iyn	0.588502	12.9374	1.33963	185 186
1j77	0.626947	22.0004	2.32819	136 137
**1n97**	**0.72756**	**1.64582**	**16.1534**	**98 222 223**
**1u5u**	**0.644717**	**1.29679**	**8.21622**	**67 323**
2fdv	0.747632	1.28909	1.51184	372 433 437
**2fmy**	**0.688978**	**3.09737**	**16.7876**	**43 82**
2nwb	0.634761	9.01587	4.88908	132 238
2wiy	0.603125	21.6574	2.67983	360 361
3bnj	0.902126	1.34137	1.83765	171 214 215
**3e4w**	**0.652893**	**1.47317**	**9.01197**	**28 83**
3h4n	0.8548	1.64944	1.2448	20 30
**3o72**	**0.761116**	**1.90662**	**8.11691**	**334 336**
3qm9	0.59516	15.0392	2.25906	10 115
**4b2n**	**0.82127**	**1.77885**	**19.2441**	**388 393 641**
4eic	0.80378	1.66497	1.29697	13 14 68
4iam	0.595079	1.75125	1.62449	83 112
4u9b	0.626374	10.2829	1.91077	137 138

Table S5: **Comparison of Ligsite-csc and our method at the "HEM" binding site.** The table consists of 21 structures containing "HEM" binding sites, the HEM binding sites that Ligsite-csc fails to detect but our method successfully predicts are labelled in bold (4 Å cut-off).

[1]Top score of out method.

[2]The nearest distance between the top score grid assigned by our method and the ligand.

[3]The nearest distance between the top score grid assigned by Ligsite-csc and the ligand.

[4]Residue number of ligand-prefer Ramachandran box residues that are positioned less than 6 Å from the top h_score grid.
